# 3,3′,5,5′-Tetrabromobiphenyl (BB-80) and Its Hydroxylation Product (OH-BB-80) Mediate Immunotoxicity and Inhibit Embryonic Development in Zebrafish (*Danio rerio*) via the TLR4/NF-κB Signaling Pathway

**DOI:** 10.3390/toxics13040293

**Published:** 2025-04-10

**Authors:** Yongjian Shao, Yinan Zhang, Xiaofang Zhang, Yu Han, Zhiquan Liu, Jiafeng Ding, Binhao Wang, Hangjun Zhang

**Affiliations:** 1 College of Engineering, Hangzhou Normal University, Hangzhou 311121, China; 2023111010100@stu.hznu.edu.cn (Y.S.); zhangyinan98@163.com (Y.Z.); 13588028414@163.com (X.Z.); yu-han@hznu.edu.cn (Y.H.); zqliu@hznu.edu.cn (Z.L.); djf101@hznu.edu.cn (J.D.); wangbh@hznu.edu.cn (B.W.); 2 Zhejiang Provincial Key Laboratory of Wetland Intelligent Monitoring and Ecological Restoration, Hangzhou 311121, China; 3 State Environmental Protection Key Laboratory of Environmental Health Impact Assessment of Emerging Contaminants, Shanghai Academy of Environment Sciences, Shanghai 200233, China; 4 Hangzhou Internation Urbanology Research Center, Hangzhou 311121, China

**Keywords:** immunotoxicity, inflammation, oxidative stress, TLR4/NF-κB, zebrafish

## Abstract

Polybrominated biphenyls (PBBs) are metabolically transformed into monohydroxylated PBBs (OH-PBBs) in the environment and living organisms. Although OH-PBBs pose a significant health threat to organisms, little is known about the immunotoxicity of OH-PBBs. Therefore, the objectives of this study were to validate BB-80 and OH-BB-80 induced immunotoxicity and to explore the associated pathway mechanisms. Early development of zebrafish (*Danio rerio*) larvae was inhibited by 10 μg/L BB-80 and OH-BB-80, as indicated by negative changes in developmental indices. BB-80 and OH-BB-80 induced oxidative stress, significantly up-regulated reactive oxygen species (ROS) and reactive nitrogen species (RNS), and activated the antioxidant enzyme system at 10 μg/L. The mRNA expression levels of inflammatory cytokines and inflammatory chemokines were up-regulated, indicative of the onset of inflammation in zebrafish after BB-80 and OH-BB-80 exposure. In addition, downregulation of toll-like receptor 4 (TLR4), MyD88, and NF-κB pathway-related genes was observed, suggesting that BB-80 and OH-BB-80 target the TLR/NF-κB signaling pathway. Molecular docking data showed that BB-80 and OH-BB-80 bound stably to TLR4. Taken together, BB-80 and OH-BB-80 mediate immunotoxicity and early developmental suppression associated with the TLR4/NF-κB signaling pathway. Our results further the understanding of BB-80- and OH-BB-80-induced immunotoxicity, highlighting the need for toxicological studies to examine the toxic effects of the transformation products of PBBs.

## 1. Introduction

Economic and industrial developments have contributed to the production and direct or indirect release of many chemicals into the environment. The toxicity associated with these chemicals has led to wide concern and is the main focus of toxicological studies. In addition, many of these chemicals can be metabolically transformed in the environment and living organisms to produce metabolites or novel contaminants distinct from the parent compound. To date, little attention has been paid to the toxic effects of metabolites, which may also affect and harm living organisms and the ecological environment. For example, N-(1,3-dimethylbutyl)-N’-phenyl-p-phenylenediamine (6PPD) quinone, a conversion product of 6PPD, is one of the main toxic substances in tire particles [1]. This study highlights the need to examine the toxic effects of the transformation products of pollutants in toxicological studies.

As common brominated flame retardants, polybrominated biphenyls (PBBs) were commonly used in a variety of products, including building materials, electronic appliances, and thermoplastics until the ban on their commercial production in the United States in 1976 [2]. However, due to their significant lipophilicity, strong bioaccumulation, and long half-life, PBBs are still detected in large quantities in various environmental systems, especially in water bodies and aquatic organisms [3]. Moreover, PBBs have been detected in human hair [4], kidney, liver, and lung [5]. As shown in the Technical Fact Sheet for Polybrominated Biphenyls (PBBs) released in the United States in 2017, various states have adopted screening values or cleanup goals for PBBs in drinking water or groundwater, ranging from 0.0001 to 5 µg/L [6]. Interestingly, the mean concentration of PBBs was found to be 0.35 ng/g in adipose tissue samples from Spanish women, while 3,3′,5,5′-tetrabromobiphenyl (BB-80) accounted for >90% of all PBBs [7]. BB-80 in the environment can be transformed into hydroxylated BB-80 (OH-BB-80) through photoformation, natural manganese oxide-catalyzed oxidation, and chloroperoxidase transformation. OH-BB-80 has been detected in a variety of marine organisms, with shark, dolphin, and whale lipids containing 0.1–7.5, 12–800, and 12–49 ng/g, respectively [8,9,10]. These studies indicated that the concentration and accumulation of BB-80 and OH-BB-80 in animals and humans through the food chain poses a significant threat to human health. Therefore, the toxic effects of BB-80 and OH-BB-80 require further elucidation.

Many studies have shown that the presence of PBBs has multiple negative impacts on ecosystem security and human health. Previous studies have confirmed that the immune system may be a sensitive target for PBBs, and PBB-153 was found to be associated with perturbations in inflammation and oxidative stress [11]. In addition, disruption of lymphocyte function and reduction in the number and percentage of peripheral blood lymphocytes in PBB-exposed Michigan dairy farmers have been reported [12]. However, the mode of action of immunotoxicity of PBBs, especially its transformation products, remains unclear. The Toll-like receptor (TLR) family plays an important role in mediating non-specific immune responses. TLR2 and TLR4 are the main members of the TLR family, which recognize pathogens or their derivatives and activate MyD88-dependent pathways that activate NF-κB. NF-κB, a major transcription factor responsible for the regulation of innate and adaptive immune responses, promotes the release of inflammatory mediators and cytokines, and activates the immune response [13,14]. The roles of TLR2 and TLR4, as well as the NF-κB pathway, in mediating immunotoxicity to PBBs and their transformations need to be further investigated.

The zebrafish (*Danio rerio*) is a common Scleractinia fish that has become an important animal model for studying embryonic development, genetics, and immune systems. Zebrafish are a versatile animal model for studying biological processes and human diseases due to their physiological characteristics, which include high light transmission, rapid development, and ease of management and reproduction. In addition, optically transparent embryos develop externally, allowing easy observation of cell and organ development. As the zebrafish genome and the regulatory mechanisms of associated proteins are highly homologous to humans, results obtained from zebrafish model studies may be applicable to humans [15]. The ease of exposure of a large number of embryos to toxicants makes zebrafish a unique model for immunotoxicological studies [16,17].

In this study, we focused on the molecular mechanisms of BB-80- and OH-BB-80-induced immunotoxicity using zebrafish as an animal model. First, we observed phenotypic changes in heart rate, swim bladder developmental status, and deformities in zebrafish exposed to BB-80 and OH-BB-80. The effects of BB-80 and OH-BB-80 exposure on the early development of zebrafish larvae were assessed using developmental indicators such as the hatching rate and survival rate. Then, we used markers of oxidative stress and inflammation to assess the effects of BB-80 and OH-BB-80 on redox homeostasis and innate immunity in vivo. Finally, we used quantitative real-time polymerase chain reaction (qRT-PCR) to examine changes in the expression patterns of toll-like receptor 2 (*TLR2*), toll-like receptor 4 (*TLR4*), and their downstream target genes following BB-80 and OH-BB-80 exposure to determine whether their immunotoxicity is mediated through the TLR/NF-κB signaling pathway. Molecular docking experiments demonstrated that BB-80 and OH-BB-80 bind to TLR4 by acting as small molecules. Our results demonstrate the effects of BB-80 and OH-BB-80 exposure on the early development and innate immune system of zebrafish and their mechanisms, and further the understanding of BB-80 and OH-BB-80-induced immunotoxicity, suggesting that the BPP transformation products have strong toxic effects on organisms. Our data also provide theoretical references for an ecological early warning indicator system, as well as protection of the ecological environment.

## 2. Materials and Methods

### 2.1. Chemicals

BB-80 (CAS: 16400-50-3, 98% purity) was purchased from Jilin Yanshin Technology Co (Jilin, China). OH-BB-80 was synthesized in the laboratory as Marsh et al. described [9]; the synthesis process is described in Text S1. Dimethyl sulfoxide (DMSO, CAS: 67-68-5, purity 99.5%) was purchased from Sigma-Aldrich (St. Louis, MI, USA). Methanol, acetonitrile, n-hexane, and isooctane were all ultra-high performance liquid chromatography-mass spectrometer grade and purchased from the Merck Group (Darmstadt, Germany). Ultrapure water was prepared by Millipore Water Systems (Millipore, Burlington, MA, USA). DEPC water and TE buffer were purchased from Thermo Fisher (Waltham, MA, USA). Yeast extract, NaCl, CaCl_2_, tryptone, agar powder, and kanamycin were purchased from Sinopharm (Beijing, China).

### 2.2. Zebrafish Maintenance and Exposure Experiment Design

Two to three pairs of six-month-old adult zebrafish (wild-type, AB strain) were placed in breeding tanks with sterile ultrapure water and allowed to freely mate, spawn, and fertilize for 1–2 h. After fertilization, the eggs were transferred to sterile incubation tanks as soon as possible and prepared for the BB-80 and OH-BB-80 exposure experiments. During the exposure experiments, the sterile E3 culture medium (containing 0.1% methylene blue) was changed every 24 h. Seven experimental groups were set up: a 0.1% DMSO control group, BB-80 (1, 3, and 10 μg/L) exposure groups, and OH-BB-80 (1, 3, and 10 μg/L) exposure groups. BB-80 and OH-BB-80 exposure concentrations are referenced to the reported environmentally relevant concentrations (up to 53.9 μg/L in water) [8,9,10,18]. The exposure times were 7, 14, and 21 days. Petri dishes containing 200 mL of BB-80- or OH-BB-80-containing solution were used in our experimental exposure system. Zebrafish embryos with normal development at 2 h post-fertilization was selected. Embryos were separated equally into 300 per petri dish for the solvent control group, and different concentration exposure groups with four dishes/treatment group. The solution was changed daily. All zebrafish experiments complied with relevant animal welfare guidelines and received research ethics approval from Hangzhou Normal University (Approval number: HSD-20240312-01) in 12 March 2024.

### 2.3. Measurement of BB-80 and OH-BB-80 Concentration Levels in the Exposure Solution

The detailed method for the extraction of BB-80 and OH-BB-80 from the exposure solution can be found in Text S2. Gas chromatography and ultra-performance liquid chromatography were used to detect changes in the concentrations of the water contaminants (BB-80 and OH-BB-80) at the beginning of the experiment and after 24 h exposure. Water samples were enriched by hydrophile-lipophile balance (HLB) solid-phase extraction cartridges (Waters, Milford, CT, USA).

### 2.4. Evaluation of Embryonic Developmental Toxicity

To determine the effects of BB-80 and OH-BB-80 exposure on the early development of zebrafish, healthy embryos were selected and placed in petri dishes containing 200 mL of 0, 1, 3, and 10 μg/L BB-80 and OH-BB-80 and incubated until 72 h postfertilization (hpf), with 100 embryos in each dish and each concentration group containing four replicates. At 24, 48, and 72 hpf postfertilization, the number of larvae hatching and developmental malformations in each group were counted, and the hatching and malformation rates were calculated. The condition of zebrafish embryos was observed under a stereoscope, and representative abnormal zebrafish embryos were selected and photographed.

### 2.5. Oxidative Stress Tests

Zebrafish homogenate was prepared by placing 0.6 g zebrafish into 3 mL PBS and grinding. The homogenate was mixed with 2 mL PBS and centrifuged for 10 min at 12,000 rpm and 4 °C. Total protein (with standard: BCA method, Catalogue: A045-3-2), reactive oxygen species (ROS) (Chemical fluorescence method, Catalogue: E004-1-1), malondialdehyde (MDA) (TBA method, Catalogue: A003-1-1), total antioxidant capacity (T-AOC) (Colorimetric method, Catalogue: A015-1-2), nitric oxide (NO) (Nitrate reductase method, Catalogue: A012-1-1), superoxide dismutase (SOD) (Hydroxylamine method, Catalogue: A001-1-1), catalase (CAT) (Visible light, Catalogue: A007-1-1), inducible nitric oxide synthase (iNOS) (Colorimetric method, Catalogue: A014-1-2), and total nitric oxide synthase (TNOS) (Colorimetric method, Catalogue: A014-1-2) levels and activities were assessed in the supernatant using the corresponding kits (Nanjing Jiancheng Bioengineering institute, Nanjing, China).

### 2.6. Gene Expression Analysis

The total RNA of juvenile fish was extracted with Trizol reagent. We determined RNA concentration and integrity using NanoDrop (Thermo Scientific, Waltham, MA, USA) and agarose gel electrophoresis. Sample RNA was diluted to 300 ng/μL before reverse transcription. Protocols for genomic DNA contamination removal, RNA reverse transcription, and qPCR were performed using Hifair^®^ III 1st Strand cDNA Synthesis SuperMix for qPCR (gDNA digester plus) and Hieff^®^ qPCR SYBR^®^ Green Master Mix (No Rox) kits (Yisheng Bio-technology Co., Ltd., Shanghai, China). qRT-PCR was performed on a QuantStudio 7 Flex Real-Time PCR system (Applied Biosystems, Waltham, MA, USA).The primer sequences are shown in Table S1. Primers were synthesized by Shanghai Bioengineering Co. The internal reference gene was β-actin, and gene expression level was analyzed by the 2^−△△Ct^ method.

### 2.7. Cytokine Enzyme-Linked Immunosorbent Assay (ELISA)

Zebrafish were homogenized at low temperature by adding PBS (pH 7.4) at a weight:volume ratio of 1 g: 9 mL. The supernatant was collected and used for the ELISA or frozen at −20 °C. IL-1β (Catalogue: MC90018), IL-6 (Catalogue: H007-1-2), IL-10(Catalogue: H009-1-2), and TNF-α (Catalogue: MC90148) protein levels were measured by ELISA using a Fish ELISA Kit (Nanjing Jiancheng Institute of Biological Engineering, Nanjing, China). All test methods were performed according to the manufacturer’s instructions and then absorbance was measured on a TECAN SPARK 10M multifunctional enzyme marker.

### 2.8. Molecular Docking

Molecular docking was used to assess the binding of BB-80 and OH-BB-80 to TLR4. More details are described in Text S3.

### 2.9. Statistical Analysis

All data were analyzed using Original 8.0 Statistics software. Results are expressed as mean ± standard error of the mean (SEM). Homogeneity of variance and normality of all data were checked by Levene and Kolmogorov–Smirnov tests. Physiological and biochemical indices such as ROS were assessed by two-way ANOVA based on Tukey’s multiple comparisons to evaluate the effect of exposure time and exposure concentration. The rest of the analyses were performed using one-way ANOVA based on Tukey’s multiple comparisons to assess the differences between the different groups. The significance level was set at *p* < 0.05 or *p* < 0.01, where *p* < 0.05 represents a significant difference and *p* < 0.01 represents a highly significant difference.

## 3. Results and Discussion

### 3.1. Changes in the Concentration of BB-80 and OH-BB-80 in the Exposure Fluid After 24 h

**Figure 1:**
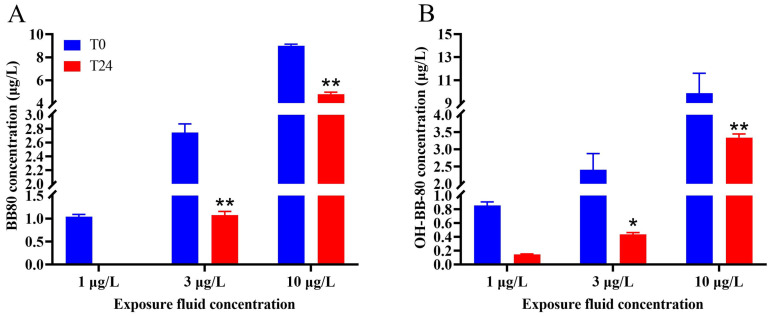
Changes in the concentration of BB-80 (**A**) and OH-BB-80 (**B**) in the exposure fluid after 0 h and 24 h. Data are expressed as mean ± SEM, *n* = 6 per group, * *p* < 0.05,** *p* < 0.01.

GC and UPLC were used to detect changes in the concentrations of contaminants in the water between the beginning of the experiment and after 24 h exposure. We found that the true average concentrations of the 1, 3, and 10 μg/L BB-80 exposure groups were 1.04, 2.75, and 9.01 μg/L, respectively, with recoveries of 90–104%. After 24 h exposure, the concentration of BB-80 in the water decreased to ND, 1.07, and 4.80 μg/L, respectively, with the 3 μg/L and 10 μg/L exposure groups showing highly significant decreases in concentration levels (*p* < 0.01; Figure 1A). The true mean concentrations of the 1, 3, and 10 μg/L OH-BB-80 exposure groups were 0.86, 2.41, and 9.87 μg/L, respectively, with recoveries of 81–99%. After 24 h exposure, the concentration of OH-BB-80 in the water decreased to 0.15, 0.44, and 3.34 μg/L, respectively, with the 3 μg/L and 10 μg/L exposure groups showing significant decreases in concentration levels (*p* < 0.05; Figure 1B). These results indicated that BB-80 and OH-BB-80 were absorbed by zebrafish.

### 3.2. BB-80 and OH-BB-80 Inhibit Early Growth and Development in Zebrafish

Changes in developmental markers such as hatching rate, survival rate, and malformation rate during embryonic development are good indicators of developmental toxicity induced by environmental toxic stress [19,20,21]. In this study, we systematically evaluated the effects of BB-80 and OH-BB-80 on the early growth and development of zebrafish by measuring embryonic malformation efficiency, hatching efficiency, survival efficiency, heart rate, and swim bladder developmental status after exposure.

**Figure 2:**
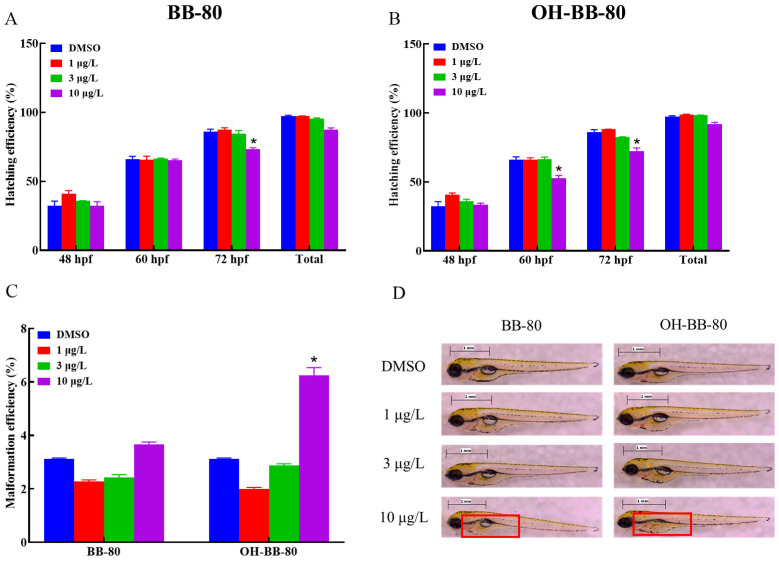
Hatching efficiency (**A**,**B**), malformation efficiency (**C**), and swim bladder developmental status (**D**) of zebrafish exposed to BB-80 and OH-BB-80. The red box in D shows the state of the swim bladder. Data are expressed as mean ± SEM, n = 6 per group, * *p* < 0.05.

BB-80 and OH-BB-80 significantly affected the embryonic development of zebrafish (Figure 2A,B). Heart rate is an important indicator of early embryonic growth and development. Here, the hatching rate of zebrafish embryos exposed to 10 μg/L OH-BB-80 decreased by 20% and 17% at 60 and 72 hpf, respectively, while exposure to 10 μg/L BB-80 led to a decrease of 18% at 72 hpf. In addition, OH-BB-80 displayed extremely strong teratogenic effects (Figure 2C) with a significantly higher (94%) embryo malformation rate in the 10 μg/L OH-BB-80 group compared to the control group. Interestingly, 1 μg/L and 3 μg/L BB-80 and OH-BB-80 exposed groups showed a decrease in malformation rate and an increase in hatching rate, although it did not show significance (*p* > 0.05). This may indicate the dimorphism of BB-80 and OH-BB-80 concentrations in inducing developmental growth. Furthermore, exposure to BB-80 and OH-BB-80 led to a concentration-dependent downward trend in zebrafish survival efficiency, although this was not statistically significant (*p* > 0.05). Administration of PBBs during early gestation in rats reportedly causes reduced offspring survival and increased embryonic malformations, which is consistent with the results of the present study [22]. Together, our findings suggest that BB-80 and OH-BB-80 interfere with zebrafish embryonic development. Notably, the swim bladders of zebrafish in the OH-BB-80 10 μg/L group did not swell (Figure 2D), which is a phenomenon known as “non-inflated swim bladders”. However, this phenomenon did not occur in zebrafish exposed to BB-80. Based on our findings that OH-BB-80, but not BB-80, exhibited teratogenic effects on zebrafish, we speculate that OH-BB-80 may be more developmentally toxic to zebrafish than BB-80, although further studies are required to confirm this. In conclusion, our findings provide convincing evidence that BB-80 and OH-BB-80 inhibit early zebrafish growth and development, and that OH-BB-80 exhibits greater developmental toxicity than BB-80.

### 3.3. BB-80 and OH-BB-80 Induce Oxidative Stress in Juvenile Zebrafish

**Figure 3:**
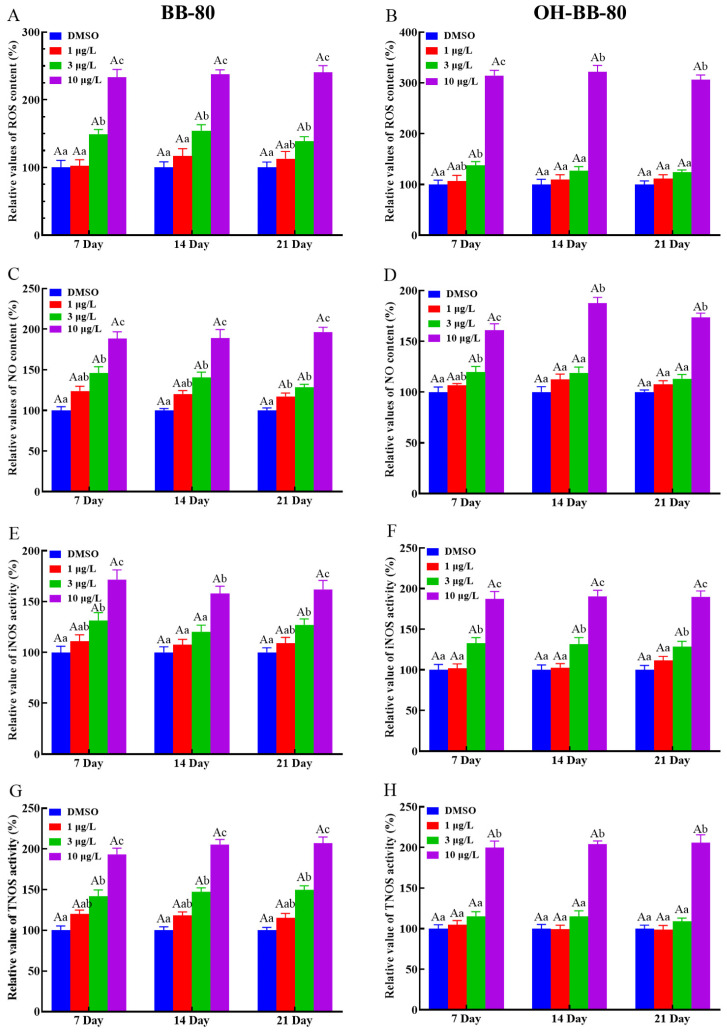
Relative ROS and NO content, as well as iNOS and TNOS activities, in zebrafish exposed to BB-80 (**A**,**C**,**E**,**G**) and OH-BB-80 (**B**,**D**,**F**,**H**). Data are expressed as mean ± SEM, n = 6 per group, superscript lower-case letters indicate significant differences in concentration groups at the same exposure cycle (*p* < 0.05) and superscript capital letters indicate significant differences between exposure cycles (*p* < 0.05).

Exposure to toxic chemicals may lead to an imbalance in the production and consumption of ROS and reactive nitrogen, which can cause oxidative stress and oxidative damage in organisms [23]. Here, we assessed the levels of redox indicators such as ROS levels, NO content, and NOS activity to determine whether oxidative stress occurred in zebrafish larvae exposed to BB-80 and OH-BB-80. We found that ROS levels in zebrafish larvae were increased in a dose-dependent manner following exposure to BB-80 and OH-BB-80 (Figure 3A,B), consistent with the study by Shi et. al. [24]. Specifically, the ROS content in zebrafish larvae exposed to 10 μg/L BB-80 and OH-BB-80 was significantly higher than the control groups at all three time points (*p* < 0.01). Similarly, BB-80 and OH-BB-80 exposure led to significant increases in NO production in zebrafish larvae with the most significant changes occurring after exposure to 10 μg/L (*p* < 0.01; Figure 3C,D). NOS catalyzes the oxidation of guanidinium nitrogen from L-arginine into NO and L-citrulline [25]^.^ Here, we found that iNOS and TNOS activities were significantly higher in the BB-80 and OH-BB-80 exposure groups compared to the control groups (*p* < 0.01; Figure 3E–H), further corroborating the effects of BB-80 and OH-BB-80 on NO induction. Reactive oxygen radicals and reactive nitrogen radicals induce oxidative stress, which causes physiological and pathological responses in cells and tissues. Our findings indicate that exposure to BB-80 and OH-BB-80 may induce an imbalance in ROS in zebrafish.

**Figure 4:**
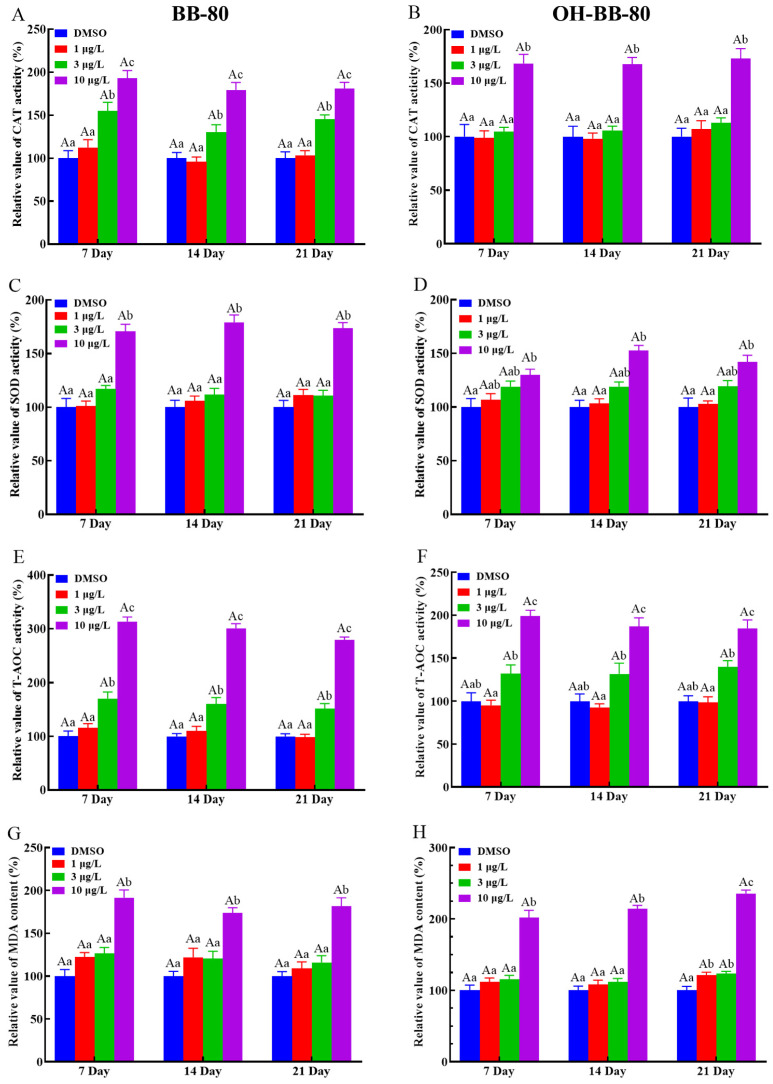
Relative CAT and SOD activities, as well as T-AOC and MDA levels, in zebrafish exposed to BB-80 (**A**,**C**,**E**,**G**) and OH-BB-80 (**B**,**D**,**F**,**H**). Data are expressed as mean ± SEM, n = 6 per group, superscript lower-case letters indicate significant differences in concentration groups at the same exposure cycle (*p* < 0.05) and superscript capital letters indicate significant differences between exposure cycles (*p* < 0.05).

We next used antioxidant markers to determine whether oxidative stress occurs in zebrafish. Antioxidant enzymes such as SOD and CAT play an important role in protecting against ROS damage and have been widely used as biomarkers for evaluating oxidative stress induced by environmental chemicals [20,26,27]. Here, we found that CAT (Figure 4A,B) and SOD (Figure 4C,D) activities were significantly increased (*p* < 0.01) in both the 10 μg/L BB-80 and OH-BB-80 exposure groups compared to the control group at the three different exposure times. Interestingly, we found that CAT activity levels increased much earlier than SOD at BB-80 and OH-BB-80 concentrations of 3 μg/L. This may be due to the relationship between SOD and CAT. As a free radical scavenger, SOD reacts rapidly to ROS generated by toxic chemicals, effectively converting the highly reactive superoxide radicals (O_2_^−^) to hydrogen peroxide (H_2_O_2_) [28], while CAT subsequently participates in the defense against oxidants by detoxifying H_2_O_2_ [29]. T-AOC levels reflect the T-AOC of the various antioxidant and antioxidant enzyme compositions. As shown in Figure 4E,F, a significant increase in T-AOC was observed in zebrafish exposed to 10 μg/L BB-80 and OH-BB-80 at all three time points. Moreover, the T-AOC in zebrafish larvae increased in a dose-dependent manner following exposure to BB-80 and OH-BB-80. Our findings indicate that following exposure to BB-80 and OH-BB-80, zebrafish activate the antioxidant system in vivo and undergo a strong antioxidant response.

We next examined the presence of oxidative stress in BB-80- and OH-BB-80-exposed zebrafish from the perspective of damage caused by oxidative stress. ROS react with lipids, DNA, and proteins in organisms, causing oxidative damage such as lipid peroxidation, protein denaturation, and gene mutations, which lead to physiological and pathological responses in cells and tissues [30]. Here, we assessed MDA levels to evaluate lipid peroxidation levels in zebrafish larvae, as an indirect measure of oxidative stress levels induced by BB-80 and OH-BB-80. MDA levels were significantly increased in zebrafish larvae continuously exposed to BB-80 and OH-BB-80 at concentrations of 10 μg/L (Figure 4G,H).

Changes in all of the above indicators suggest that oxidative stress occurred in zebrafish and showed a clear concentration dependence. However, there were no significant differences between exposure cycles, and the levels of BB-80 and OH-BB-80-induced oxidative stress may be time-independent and need to be investigated over longer life-cycle exposures. Thus, our findings demonstrate that exposure to BB-80 and OH-BB-80 induces an increase in ROS content and reactive nitrogen radical levels in zebrafish larvae, which causes an imbalance in the redox state, and in turn induces oxidative stress resulting in oxidative damage, such as lipid peroxidation. In response to increased ROS, the antioxidant system of the juvenile zebrafish is activated in order to eliminate the ROS generated in the body. How BB-80 and OH-BB-80 induce the onset of oxidative stress requires further elucidation.

### 3.4. BB-80 and OH-BB-80 Trigger Inflammatory Responses in Juvenile Zebrafish

**Figure 5:**
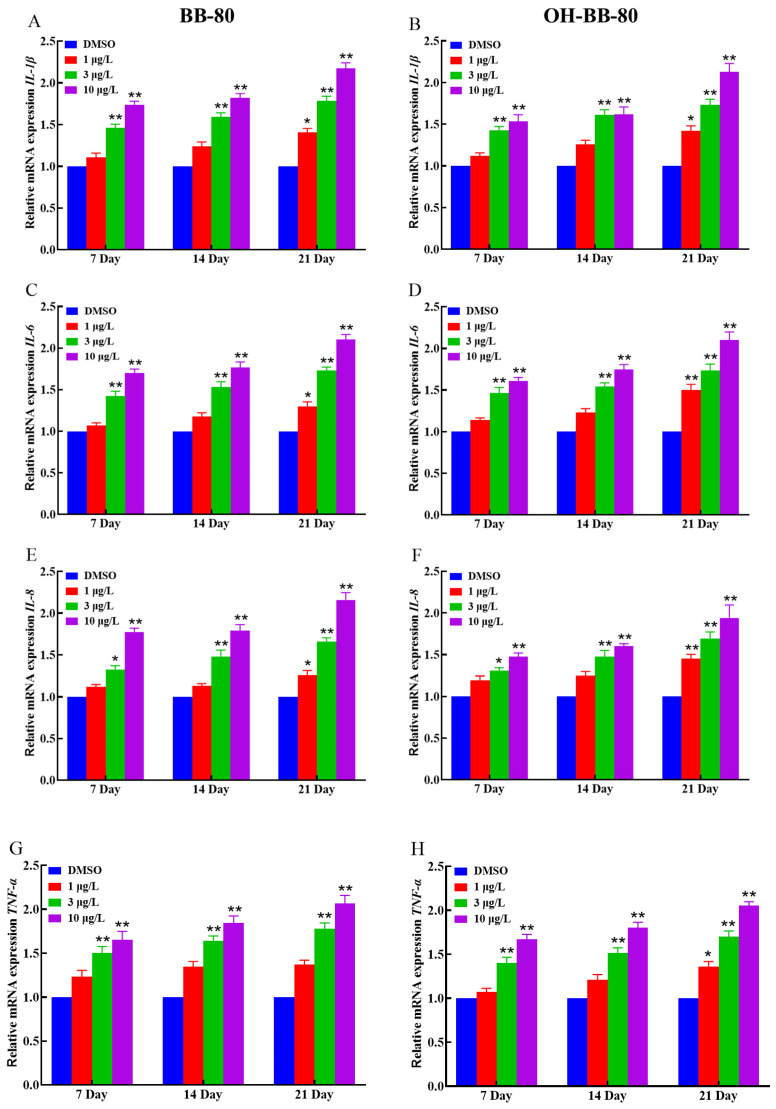
Relative *IL-1β*, *IL-6*, *TNF-α*, and *IL-8* mRNA expression levels in zebrafish exposed to BB-80 (**A**,**C**,**E**,**G**) and OH-BB-80 (**B**,**D**,**F**,**H**). Data are expressed as mean ± SEM, n = 6 per group, * *p* < 0.05, ** *p* < 0.01.

Inflammation and oxidative stress are mutually reinforcing, with inflammation increasing ROS production, which in turn exacerbates inflammation [31]. Based on our oxidative stress data, we hypothesized that an inflammatory response also occurred in zebrafish following BB-80 and OH-BB-80 exposure. Thus, we next measured the expression levels of inflammatory cytokines and chemokines to determine whether an inflammatory response occurred in the zebrafish in response to BB-80 and OH-BB-80 exposure. Inflammatory cytokines are markers of inflammation. As shown in Figure 5A–H, BB-80 and OH-BB-80 significantly induced the expression of inflammatory cytokines. *IL-1β* (Figure 5A,B) and *IL-6* (Figure 5C,D) mRNA levels were significantly increased (*p* < 0.01) in zebrafish following exposure to 3 and 10 μg/L BB-80 and OH-BB-80. A similar trend was observed for *IL-8* (Figure 5E,F) and *TNF-α* (Figure 5G,H) mRNA expression levels. Specifically, after 21 days of exposure to 3 μg/L and 10 μg/L BB-80 and OH-BB-80, *IL-8* and *TNF-α* mRNA levels were significantly higher than the DMSO control group (*p* < 0.05). Furthermore, IL-8 and TNF-α mRNA expression levels showed a positive correlation with the concentration of BB-80 and OH-BB-80. In addition, *IL-6* and *IL-8* mRNA expression levels were found to be significantly higher in the 1 μg/L concentration group than the DMSO control group at longer exposure times, with the OH-BB-80 exposure group (*p* < 0.05) showing a more significant difference than BB-80 exposure group (*p* < 0.01).

**Figure 6:**
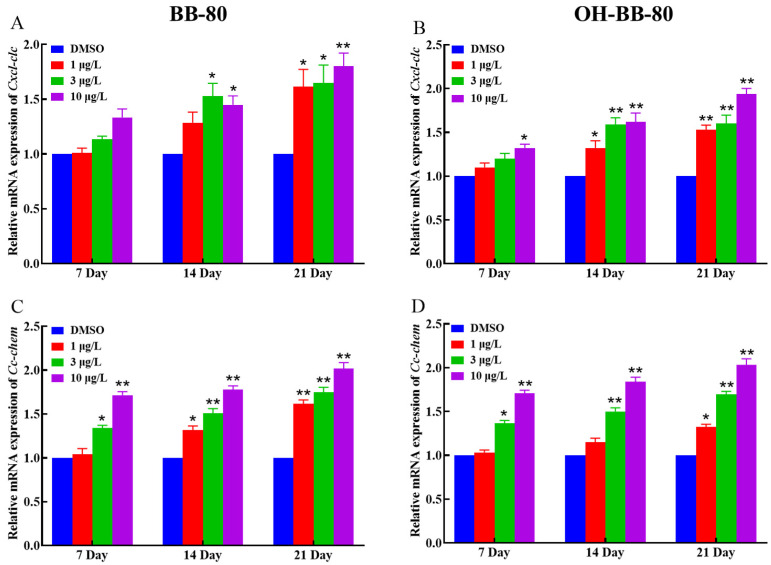
Relative *Cxcl-clc* and *Cc-chem* mRNA expression levels in zebrafish exposed to BB-80 (**A**,**C**) and OH-BB-80 (**B**,**D**). Data are expressed as mean ± SEM, n = 6 per group, * *p* < 0.05, ** *p* < 0.01.

Inflammatory chemokines play a crucial role in controlling leukocyte recruitment in the inflammatory response [32]. Next, we examined changes in the mRNA expression levels of the chemokines *Cxcl-clc* and *Cc-chem* following BB-80 and OH-BB-80 exposure. We found that the expression of *Cxcl-clc* and *Cc-chem* was significantly correlated with exposure to BB-80 and OH-BB-80, and that there was a significant dose effect (Figure 6A–D). This trend is consistent with the expression of inflammatory cytokines. Our findings indicated that exposure to BB-80 and OH-BB-80 stimulates a significant increase in the expression levels of inflammatory cytokines and chemokines, leading to an imbalance in immune response homeostasis and triggering an inflammatory response. However, the mechanism through which BB-80 and OH-BB-80 cause inflammation remains unclear.

### 3.5. BB-80 and OH-BB-80 Activate the TLR4/NF-κB Innate Immune Signaling Pathway to Induce an Inflammatory Response

We further investigated the specific mechanisms through which BB-80 and OH-BB-80 induce inflammatory responses and oxidative stress. NF-κB is a nuclear transcription factor that consists of five subunits, p50, p52, RelA/p65, cRel, and RelB, which regulate the transcription of related genes by binding to DNA-specific sequences [33]. NF-κB plays an important role in regulating oxidative stress, inflammation, cell cycle, and apoptosis. In the context of oxidative stress, activation of NF-κB inhibits expression of cardiac mitochondrial respiratory chain complexes II, III, IV, and V, leading to mitochondrial respiratory chain dysfunction and ROS production [34]. TLRs are well-characterized in fish and depend on the TLR/NF-κB pathway for signaling [35]. The TLR/NF-κB pathway has long been recognized as a classic pro-inflammatory pathway that promotes pro-inflammatory gene expression that influences cytokines, chemokines, and adhesion molecules [36]. TLRs are transmembrane receptors that are expressed on a variety of cell types, including immune and non-immune cells, and can be localized on the cell surface or in the endosomes [37]. TLRs bind to exogenous and potentially endogenous ligands and activate MyD88-dependent and/or MyD88-independent signaling pathways through different adaptor proteins such as MyD88, TIRAP, TRAM, and/or TRIF [38]. In the MyD88-dependent signaling pathway, MyD88 recruits IRAKs leading to their phosphorylation, and subsequent recruitment of TAK, which activates the downstream IKK/NF-κB cascade via phosphorylation, leading to the activation of NF-κB and modulation of inflammatory cytokines, inflammatory chemokines, and other immune-related genes that trigger a pro-inflammatory signaling cascade [39]. Therefore, we speculate that BB-80 and OH-BB-80 may mediate the inflammatory response and oxidative stress through the TLR/NF-κB pathway.

**Figure 7:**
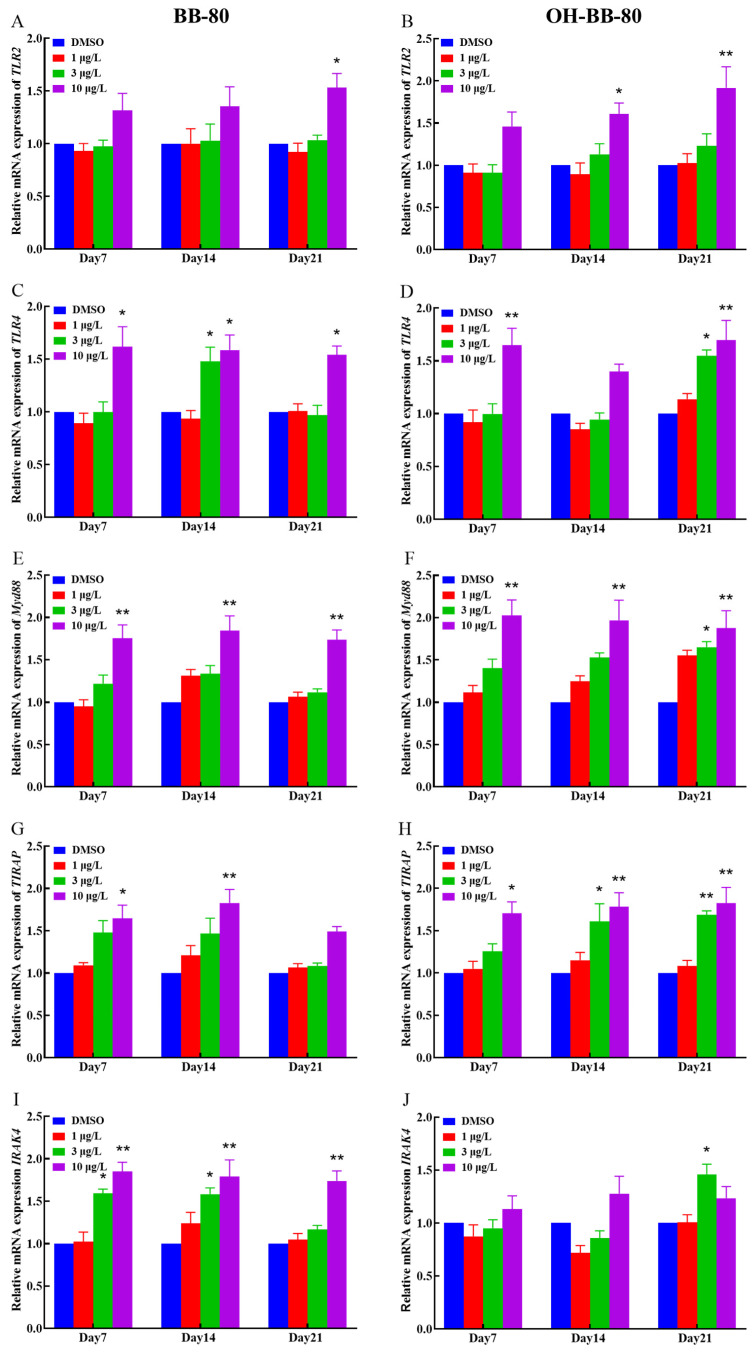

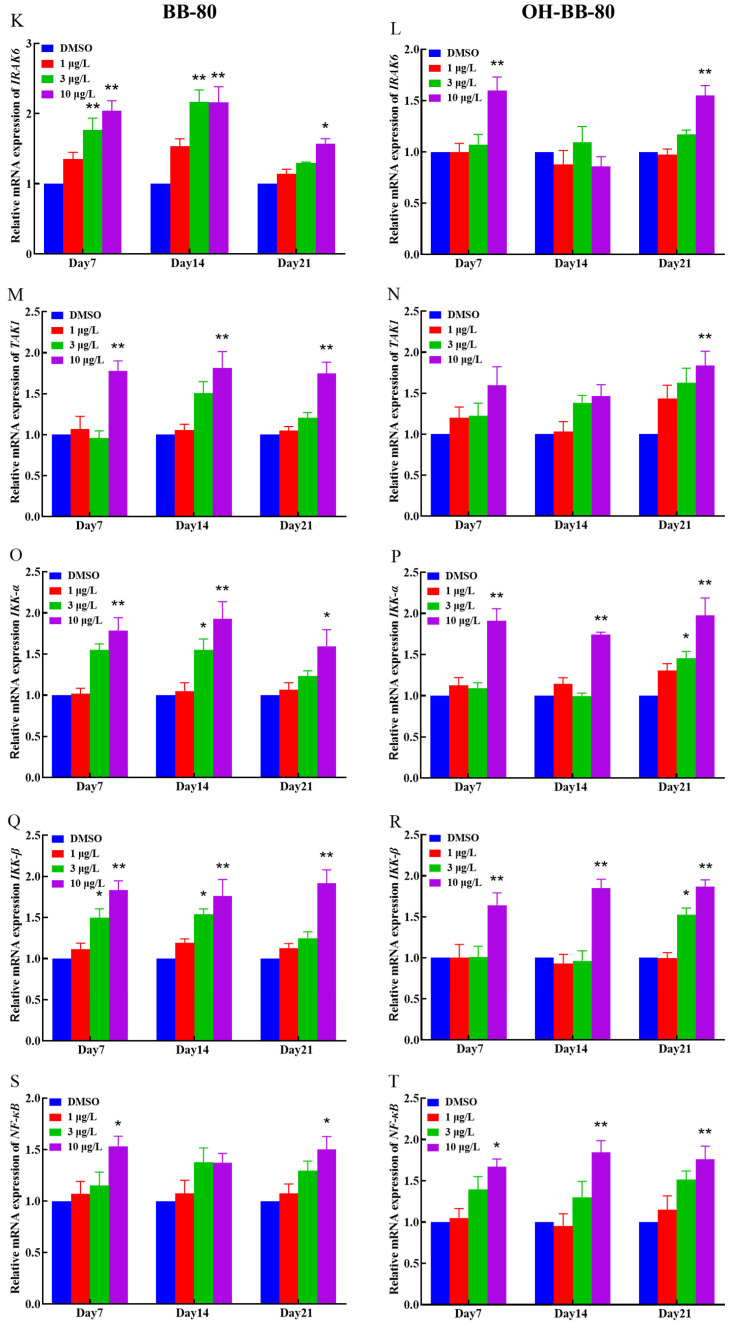
Relative *TLR2*, *TLR4*, *Myd88*, *IRAK4*, *RAK6*, *TAK1*, *IKK-α*, *IKK-β*, *TIRAP*, and *NF-κB* mRNA expression levels in zebrafish exposed to BB-80 (**A**,**C**,**E**,**G**,**I**,**K**,**M**,**O**,**Q**,**S**) and OH-BB-80 (**B**,**D**,**F**,**H**,**J**,**L**,**N**,**P**,**R**,**T**). Data are expressed as mean ± SEM, n = 6 per group, * *p* < 0.05, ** *p* < 0.01.

**Figure 8:**
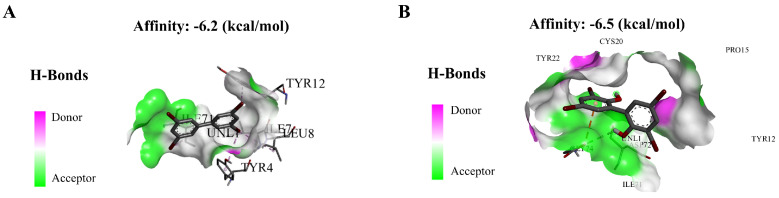
Molecular docking of BB-80 (**A**) and OH-BB-80 (**B**) with TLR4.

Binding of exogenous ligands such as peptidoglycan, lipocholic acid, and lipopolysaccharide to TLR2 or TLR4 has been reported to enhance the production of pro-inflammatory cytokines and chemokines such as IL-6 and IL-17 [40,41]. Therefore, we selected TLR2 and TLR4 as representative TLRs, and measured changes in their mRNA expression levels, as well as their downstream pathway genes. In addition, we performed molecular docking on BB-80 and OH-BB-80 to determine the specific mechanisms of BB-80- and OH-BB-80-induced inflammation and oxidative stress in juvenile zebrafish. Exposure to 10 μg/L BB-80 exposure led to significantly increased *TLR4* (Figure 7C), *Myd88* (Figure 7E), *IRAK4* (Figure 7I), *IRAK6* (Figure 7K), *TAK1* (Figure 7M), *IKK-α* (Figure 7O), and *IKK-β* (Figure 7Q) mRNA expression levels compared to the DMSO control group (*p* < 0.05). In contrast, although *TLR2* (Figure 7B), *TIRAP* (Figure 7G), and *NF-κB* (Figure 7S) mRNA expression levels were not significantly different in the pre-exposure period, significant upregulation was observed in the post-exposure period. Exposure to 10 μg/L OH-BB-80 led to a significant time-dependent increase in *Myd88* (Figure 7F), *TIRAP* (Figure 7H), *IKK-α* (Figure 7P), *IKK-β* (Figure 7R), and *NF-κB* (Figure 7T) mRNA expression levels. Although the trends in the expression levels of the remaining genes were different, all genes were eventually upregulated. Thus, our findings indicated that exposure to BB-80 and OH-BB-80 leads to a dose-dependent significant increase in the mRNA expression levels of TLR/NF-κB signaling pathway-related genes. Our results provide strong evidence that the TLR/NF-κB signaling pathway may be involved in the inflammatory response induced by exposure to BB-80 and OH-BB-80. Resmand et al. showed that TLR4 has a hydrophobic pocket, which allows TLR4 to bind to hydrophobic molecules more readily than other members of the TLR family, thereby further activating downstream pathways that trigger inflammation [42]. Therefore, we further examined the interaction of BB-80 and OH-BB-80 with TLR4 using molecular docking (Figure 8A,B). The molecular docking scores of TLR4 with BB-80 and OH-BB-80 were −6.2 and −6.5 kcal/mol, respectively, and the binding of both contaminants to TLR4 was stable. Hydrogen bonding is essential for ligand-target receptor binding; TLR4 was found to bind to OH-BB-80 and form two hydrogen bonds, whereas TLR4 binds to BB-80 but does not form hydrogen bonds. Our findings suggest that BB-80 and OH-BB-80 induce inflammation and oxidative stress in zebrafish by stimulating the TLR4/NF-κB signaling pathway.

Many studies have shown that oxidative stress and inflammatory responses are closely related to the early development of organisms. For example, 3,3′,4,4′,5-pentachlorobiphenyl (PCB126) exposure has been shown to decrease the activity of antioxidant enzymes in zebrafish, as well as induce physiological malformations [43]. Here, we found that zebrafish also displayed developmental toxicity after exposure to BB-80 and OH-BB-80, and hypothesize that this developmental toxicity is the result of oxidative stress and inflammation. However, further studies using antioxidant and inflammatory factor inhibitors are required to further elucidate the precise mechanisms of action.

## 4. Conclusions

**Figure 9:**
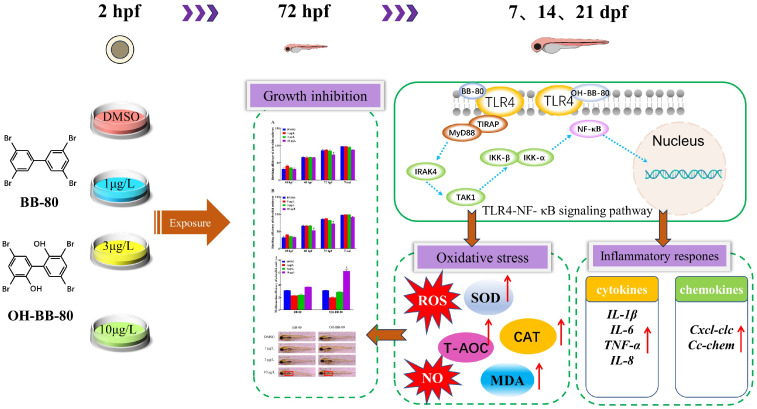
Mechanisms through which BB-80 and OH-BB-80 induce developmental toxicity and immunotoxicity in zebrafish. SOD: Superoxide dismutase. CAT: Catalase. T-AOC: Total antioxidant capacity. MDA: Malondialdehyde. ROS: reactive oxygen species. NO: Nitric oxide. Red arrows represent rising levels or content.

This study demonstrated that BB-80 and OH-BB-80 bind to TLR4, activate the TLR4/NF-κB signaling pathway, induce the expression of inflammatory cytokines and chemokines, and promote ROS production, which in turn lead to inflammatory response and oxidative stress, and ultimately mediates immunotoxicity and early developmental toxicity (Figure 9). The effects of BB-80 and OH-BB-80 exposure on the early development and innate immune system of zebrafish and their mechanisms were revealed, contributing to the understanding of BB-80 and OH-BB-80-induced immunotoxicity, and suggesting that the PBB transformation products have strong toxic effects on organisms. These data also provide theoretical references for ecological security early warning and protection of the ecological environment.

## Data Availability

The data presented in this study are available on request from the corresponding author.

## Supplementary Materials

The following supporting information can be downloaded at: https://www.mdpi.com/article/10.3390/toxics13040293/s1. Text S1. OH-BB-80 synthesis. Text S2. BB-80 and OH-BB-80 extraction [44]. Text S3. Molecular docking. Table S1. Sequences of primers [14,45,46].

## References

[1] Varshney S., Gora A.H., Siriyappagouder P., Kiron V., Olsvik P.A. (2022). Toxicological effects of 6PPD and 6PPD quinone in zebrafish larvae. J. Hazard. Mater..

[2] Wattigney W.A., Irvin-Barnwell E., Li Z., Ragin-Wilson A. (2022). Biomonitoring of toxic metals, organochlorine pesticides, and polybrominated biphenyl 153 in Michigan urban anglers. Environ. Res..

[3] Terrell M.L., Berzen A.K., Small C.M., Cameron L.L., Wirth J.J., Marcus M. (2009). A cohort study of the association between secondary sex ratio and parental exposure to polybrominated biphenyl (PBB) and polychlorinated biphenyl (PCB). Environ. Health.

[4] Zhao G., Wang Z., Dong M.H., Rao K., Luo J., Wang D., Zha J., Huang S., Xu Y., Ma M. (2008). PBBs, PBDEs, and PCBs levels in Hair of Residents Around E-Waste Disassembly Sites in Zhejiang Province, China, and Their Potential Sources. Sci. Total Environ..

[5] Ni H., Zeng H., Tao S., Zeng E. (2010). Environmental and human exposure to persistent halogenated compounds derived from e-waste in China. Environ. Toxicol. Chem..

[6] Technical Fact Sheet—Polybrominated biphenyls (PBBs) (2017). U.S. Environ. Prot. Agency. https://www.epa.gov/sites/default/files/2017-12/documents/ffrro_factsheet_pbb_11-16-17_508.pdf.

[7] Zhao H., Jiang J., Wang Y., Lehmler H.J., Buettner G.R., Quan X., Chen J. (2015). Monohydroxylated polybrominated diphenyl Ethers (OH-PBDEs) and dihydroxylated polybrominated biphenyls (Di-OH-PBBs): Novel photoproducts of 2,6-dibromophenol. Environ. Sci. Technol..

[8] Kato Y., Okada S., Atobe K., Tetsuya E., Futoshi M., Takayoshi O., Koichi H. (2009). Simultaneous determination by APCI-LC/MS/MS of hydroxylated and methoxylated polybrominated diphenyl ethers found in marine biota. Anal. Chim. Acta.

[9] Marsh G., Athanasiadou M., Athanassiadis I., Bergman A., Endo T., Haraguchi K. (2005). Identification, quantification, and synthesis of a novel dimethoxylated polybrominated biphenyl in marine mammals caught off the coast of Japan. Environ. Sci. Technol..

[10] Letcher R.J., Gebbink W.A., Sonne C., Born E.W., McKinney M.A., Dietz R. (2009). Bioaccumulation and biotransformation of brominated and chlorinated contaminants and their metabolites in ringed seals (*Pusa hispida*) and polar bears (*Ursus maritimus*) from East Greenland. Environ. Int..

[11] Susan S.H., Liang D., Robert B.H., Tan Y., Metrecia L.T., Marder M.E., Barton H., Melanie A.P., Douglas I.W., Dana B.B. (2023). Assessing Metabolic Differences Associated with Exposure to Polybrominated Biphenyl and Polychlorinated Biphenyls in the Michigan PBB Registry. Environ. Health Perspect..

[12] Bekesi J.G., James F.H., Anderson H.A., Fischbein A.S., Rom W., Wolff M.S., Selikoff I.J. (1978). Lymphocyte Function of Michigan Dairy Farmers Exposed to Polybrominated Biphenyls. Science.

[13] Wang K., Huang Y., Cheng B., Guo J., Peng Y., Zeng S., Zhang J., Lu H. (2023). Sulfoxaflor induces immunotoxicity in zebrafish (*Danio rerio*) by activating TLR4/NF-κB signaling pathway. Fish Shellfish. Immunol..

[14] Pei J., Chen S., Ke Q., Pang A., Niu M., Li N., Li J., Wang Z., Wu H., Nie P. (2025). Immune response to polystyrene microplastics: Regulation of inflammatory response via the ROS-driven NF-κB pathway in zebrafish (*Danio rerio*). Aquat. Toxicol..

[15] Amemiya C.T., Zhong T.P., Silverman G.A., Fishman M.C., Zon L. (1999). Zebrafish YAC, BAC, and PAC genomic libraries. Methods Cell Biol..

[16] Blechinger S.R., Warren J.T., Kuwada J.Y., Krone P.H. (2002). Developmental Toxicology of Cadmium in Living Embryos of a Stable Transgenic Zebrafish Line. Environ. Health Perspect..

[17] Fraysse B., Mons R., Garric J. (2006). Development of a zebrafish 4-day embryo-larval bioassay to assess toxicity of chemicals. Ecotoxicol. Environ. Saf..

[18] Wang X., Du T., Wang J., Kou H., Du X. (2019). Determination of polybrominated biphenyls in environmental water samples by ultrasound-assisted dispersive liquid-liquid microextraction followed by high-performance liquid chromatography. Microchem. J..

[19] Parlak V. (2018). Evaluation of apoptosis, oxidative stress responses, AChE activity and body malformations in zebrafish (*Danio rerio*) embryos exposed to deltamethrin. Chemosphere.

[20] Li H., Cao F., Zhao F., Yang Y., Teng M., Wang C., Qiu L. (2018). Developmental toxicity, oxidative stress and immunotoxicity induced by three strobilurins (pyraclostrobin, trifloxystrobin and picoxystrobin) in zebrafish embryos. Chemosphere.

[21] Jin Y., Liu Z., Peng T., Fu Z. (2015). The toxicity of chlorpyrifos on the early life stage of zebrafish: Asurvey on the endpoints at development, locomotor behavior, oxidative stress and immunotoxicity. Fish Shellfish. Immunol..

[22] (2010). Scientific Opinion on Polybrominated Biphenyls (PBBs) in Food. EFSA J..

[23] Valavanidis A., Vlahogianni T., Dassenakis M., Scoullos M. (2006). Molecular biomarkers of oxidative stress in aquatic organisms in relation to toxic environmental pollutants. Ecotoxicol. Environ. Saf..

[24] Shi X., Zhou B. (2010). The Role of Nrf2 and MAPK Pathways in PFOS-Induced Oxidative Stress in Zebrafish Embryos. Toxicol. Sci..

[25] Shnayder N.A., Petrova M.M., Moskaleva P.V., Shesternya P.P., Pozhilenkova E.A., Nasyrova R.F. (2021). The Role of Single Nucleotide Variants of NOS1, NOS2, and NOS3 Genes in the Development of the Phenotype of Migraine and Arterial Hypertension. Brain Sci..

[26] Jin Y., Zhang X., Shu L., Chen L., Sun L., Qian H., Liu W., Fu Z. (2010). Oxidative stress response and gene expression with atrazine exposure in adult female zebrafish (*Danio rerio*). Chemosphere.

[27] Shi X., Gu A., Ji G., Li Y., Di J., Jin J., Hu F., Long Y., Xia Y., Lu C. (2011). Developmental toxicity of cypermethrin in embryo-larval stages of zebrafish. Chemosphere.

[28] Maranhao H.M., Vasconcelos C.F., Rolim L.A., Neto P.J., Neto J.C., Filho R.C., Fernandes M.P., Costa-Silva J.H., Araújo A.V., Wanderley A.G. (2014). Hepatoprotective Effect of the Aqueous Extract of Simarouba amara Aublet (Simaroubaceae) Stem Bark against Carbon Tetrachloride (CCl4)-Induced Hepatic Damage in Rats. Molecules.

[29] Mansuri M.S., Jadeja S.D., Singh M., Laddha N.C., Dwivedi M., Begum R. (2017). The catalase gene promoter and 5′-untranslated region variants lead to altered gene expression and enzyme activity in vitiligo. Br. J. Dermatol..

[30] Zhang L., Dong L., Liao H. (2021). Survival strategies of bacteria in response to excessive reactive oxygen species: A review. Microbiol. China.

[31] Mittal M., Siddiqui M.R., Tran K., Reddy S.P., Malik A.B. (2014). Reactive oxygen species in inflammation and tissue injury. Antioxid. Redox Signal..

[32] Tisoncik J.R., Korth M.J., Simmons C.P., Farrar J., Martin T.R., Katze M.G. (2012). Into the eye of the cytokine storm. Microbiol. Mol. Biol. Rev..

[33] Mulero M.C., Huang D.B., Nguyen H.T., Wang V.Y., Li Y., Biswas T., Ghosh G. (2017). DNA-binding affinity and transcriptional activity of the RelA homodimer of nuclear factor κB are not correlated. J. Biol. Chem..

[34] Katare P.B., Nizami H.L., Paramesha B., Dinda A.K., Banerjee S.K. (2020). Activation of toll like receptor 4 (TLR4) promotes cardiomyocyte apoptosis through SIRT2 dependent p53 deacetylation. Sci. Rep..

[35] Kumar V. (2021). Toll-Like Receptors in Adaptive Immunity. Handbook of Experimental Pharmacology.

[36] Lawrence T. (2009). The nuclear factor NF-κB pathway in inflammation. Cold Spring Harb. Perspect. Biol..

[37] Loo Y.M., Gale M. (2011). Immune signaling by RIG-I-like receptors. Immunity.

[38] Ibsen M.S., Gad H.H., Andersen L.L., Hornung V., Julkunen I., Sarkar S.N., Hartmann R. (2015). Structural and functional analysis reveals that human OASL binds dsRNA to enhance RIG-I signaling. Nucleic Acids Res..

[39] Jacobs J.L., Coyne C.B. (2013). Mechanisms of MAVS regulation at the mitochondrial membrane. J. Mol. Biol..

[40] Chovanova L., Vlcek M., Krskova K., Penesova A., Radikova Z., Rovensky J., Cholujova D., Sedlak J., Imrich R. (2013). Increased production of IL-6 and IL-17 in lipopolysaccharide-stimulated peripheral mononuclears from patients with rheumatoid arthritis. Gen. Physiol. Biophys..

[41] Tang C.H., Hsu C.J., Yang W.H., Fong Y.C. (2010). Lipoteichoic acid enhances IL-6 production in human synovial fibroblasts via TLR2 receptor, PKCdelta and c-Src dependent pathways. Biochem. Pharmacol..

[42] Resman N., Oblak A., Gioannini T., Weiss J., Jerala R. (2014). Tetraacylated lipid A and paclitaxel-selective activation of TLR4/MD-2 conferred through hydrophobic interactions. J. Immunol..

[43] Liu H., Nie F.H., Lin H.Y., Ma Y., Ju X.H., Chen J.J. (2016). Developmental toxicity, oxidative stress, and related gene expression induced by dioxin-like PCB126 in zebrafish (*Danio rerio*). Environ. Toxicol..

[44] Zhang X., Sun Y., Gao Y. (2022). Thyroid dysfunction of zebrafish (*Danio rerio*) after early-life exposure and discontinued exposure to tetrabromobiphenyl (BB-80) and OH-BB-80. Environ. Sci. Technol..

[45] Ni A., Fang L., Xi M. (2024). Neurotoxic effects of 2-ethylhexyl diphenyl phosphate exposure on zebrafish larvae: Insight into inflammation-driven changes in early motor behavior. Sci. Total Environ..

[46] Wu Y., Wang Y., Tong Z. (2024). Pyraclostrobin induces developmental toxicity and cardiotoxicity through oxidative stress and inflammation in zebrafish embryos. Environ. Pollut..

