# Supplementary material for: 3,3′,5,5′-Tetrabromobiphenyl (BB-80) and Its Hydroxylation Product (OH-BB-80) Mediate Immunotoxicity and Inhibit Embryonic Development in Zebrafish (Danio rerio) via the TLR4/NF-κB Signaling Pathway
Source: Toxics. 2025 Apr 10;13(4):293. doi: 10.3390/toxics13040293 (PMC12030901; doi:10.3390/toxics13040293)
Supplement: Supplementary file 1 [file toxics-13-00293-s001.zip › toxics-3535892-supplementary.pdf]

Supplementary Materials

Text S1. OH-BB-80 synthesis

2,2'-Biphenol (0.19 g, 1.0 mmol) was dissolved in a mixture of dichloromethane (50 mL) and methanol (20 mL) and BTMA Br3 (1.95 g, 5.0 mmol) was added at room temperature. The solvent mixture was concentrated and extracted several times with diethyl ether. The extract was pooled and concentrated and purified on a silica gel column using ethyl acetate/hexane (3:2) as mobile phase.

Text S2. BB-80 and OH-BB-80 extraction

The actual concentrations of BB-80 and OH-BB-80 were determined in triplicate samples at the time of the fresh change of exposure solution (T0) and the next change of exposure solution (T24). The extraction methods of BB-80 and OH-BB-80 were referred to the previous research work in our laboratory [2].

Text S3. Molecular docking

The structures of Zebrafish (*Danio rerio*) TLR4 and TLR2 were not available from the Protein Data Bank (http://www.rcsb.org/), so homology modeling was used to simulate the structural models of these two proteins. Gene sequences were obtained from the NCBI database (https://www.ncbi.nlm.nih.gov/), and the gene sequences were converted to amino acid sequences encoding proteins using ORFfinder (https://www.ncbi.nlm.nih.gov/orffinder/). The obtained amino acid sequences were then compared using BLAST search (blastp) (https://blast.ncbi.nlm.nih.gov). The protein 3D structure was constructed by homology modeling strategy using the alignment pattern of SWISS-MODEL S9. The 3D structures of BB-80 and OH-BB-80 were downloaded from PubChem (https://pubchem.ncbi.nlm.nih.gov/) by CAS number. Prior to molecular docking, the ligand binding pockets of the proteins were predicted by PlayMolecule (https://www.playmolecule.com/) and ligands and receptors were prepared using AutoDock Tools 1.5.7 software. In the preparation of proteins, water molecules and other ligands are removed and hydrogen atoms and Coleman charges are added. Molecular docking between ligand and receptor was performed on AutoDock Vina. For each docking, 10 independent docking runs were performed and the binding mode with the lowest binding energy was selected for analysis. Docking results with the lowest docking energy were visualized and analyzed via Discovery Studio Visualizer 2021 Client (San Diego, CA, U.S.A.).

Table S1. Sequences of primers

Gene	Forward primer (5'-3')	Reverse primer (5'-3')	Reference
IL-1β	GGCTGTGTGTTTGGGAATCT	TGATAAACCAACCGGGACA	[3]
IL-6	TCAACTTCTCCAGCGTGATG	TCTTTCCCTCTTTTCCTCCTG	[3]
IL-8	GTGAAGCTCTACCTCCACCG	AGGACGCATGCTTTTGAAAC	[1]
TNF-α	GCTGGATCTTCAAAGTCGGGTGTA	TGTGAGTCTCAGCACACTTCCATC	[4]
Cxcl-clc	TCGCGGTAGTTTACGTCCAG	AGACATCTTCAGCGAGTCGG	[4]
Cc-chem	TGCAGCTCAACCAGAAGATG	CTTTGACGCATGGAGGATTT	[4]
TLR2	ACTGCCAGATGGGAAATT	CACCTGCCTCCAAGTAAA	[1]
TLR4	ACAGATCACCTGGACAGCAAG	TGCTTGAAAGTCCCGCATGT	[4]
Myd88	TGGACTTCACGTACCTGGAG	AGCGGTTTCCTCTGTTGTCT	[3]
TIRAP	AGTCACTGCTGGCTGCTG	TGAGACATCGGGCCCTCA	[3]

IRAK4	AGAGGACGCATGGGGGAT	CAGCCCAACACACGCAAC	[3]
IRAK6	CCTAGCCCTCCACCACCT	CCTCAAACGGCCAGCTCA	[3]
TAK1	CCTCCGCCGATATGCTGG	CCAAATGCCCCTCTGCCA	[3]
NF-κB	GCAAGATGAGAACGGAGACAC	CTACCAGCAATCGCAAACAA	[3]
IKK-α	GCGAAATAAAGGTCCGAAGG	AGCAAATCAAGCTCCCCCATA	[3]
IKK-β	ATCGGCGAGCAGATGAGC	GCAGAGGACGTTTGGGCT	[3]
β-actin	CTGTAGCCTCTCTCGGTCAG	CCATGTACGTTGCCATCCAG	[3]

References

1. Pei, J.; Chen, S.; Ke, Q.; Pang, A.; Niu, M.; Li, N.; Li, J.; Wang, Z.; Wu, H.; Nie, P. Immune response to polystyrene microplastics: Regulation of inflammatory response via the ROS-driven NF-κB pathway in zebrafish (*Danio rerio*). *Aquat. Toxicol.* **2025**, *282*, 107308.
2. Zhang, X.; Sun, Y.; Gao, Y.; Liu, Z.; Ding, J.; Zhang, C.; Liu, W.; Zhang, H.; Zhuang, S. Thyroid dysfunction of zebrafish (*Danio rerio*) after early-life exposure and discontinued exposure to tetrabromobiphenyl (BB-80) and OH-BB-80. *Environ. Sci. Technol*. **2022**, 56, 2519–2528
3. Ni, A.; Fang, L.; Xi, M.; Li, J.; Qian, Q.; Wang, Z.; Wang, X.; Wang, H.; Yan, J. Neurotoxic effects of 2-ethylhexyl diphenyl phosphate exposure on zebrafish larvae: Insight into inflammation-driven changes in early motor behavior. *Sci. Total Environ*. **2024**, 915, 170131
4. Wu, Y.; Wang, Y.; Tong, Z.; Xie W.; Wang, A.; Song, C.; Yao, W.; Wang, J. Pyraclostrobin induces developmental toxicity and cardiotoxicity through oxidative stress and inflammation in zebrafish embryos. *Environ. Pollut.* **2024**, *358*, 124490.
